# Repurposing the Electron Transfer Reactant Phenazine Methosulfate (PMS) for the Apoptotic Elimination of Malignant Melanoma Cells through Induction of Lethal Oxidative and Mitochondriotoxic Stress

**DOI:** 10.3390/cancers11050590

**Published:** 2019-04-27

**Authors:** Anh B. Hua, Rebecca Justiniano, Jessica Perer, Sophia L. Park, Hui Li, Christopher M. Cabello, Georg T. Wondrak

**Affiliations:** Department of Pharmacology and Toxicology, College of Pharmacy & Arizona Cancer Center, University of Arizona, Tucson, AZ 85724, USA; anhhua@email.arizona.edu (A.B.H.); justiniano@pharmacy.arizona.edu (R.J.); jperer@email.arizona.edu (J.P.); slpark@email.arizona.edu (S.L.P.); huili@pharmacy.arizona.edu (H.L.); cabello@pharmacy.arizona.edu (C.M.C.)

**Keywords:** reactive oxygen species, cancer, redox cycler, melanoma, oxidative stress, experimental therapeutic, phenazine methosulfate

## Abstract

Redox-directed pharmacophores have shown potential for the apoptotic elimination of cancer cells through chemotherapeutic induction of oxidative stress. Phenazine methosulfate (PMS), a N-alkylphenazinium cation-based redox cycler, is used widely as an electron transfer reactant coupling NAD(P)H generation to the reduction of tetrazolium salts in biochemical cell viability assays. Here, we have explored feasibility of repurposing the redox cycler PMS as a superoxide generating chemotherapeutic for the pro-oxidant induction of cancer cell apoptosis. In a panel of malignant human melanoma cells (A375, G361, LOX), low micromolar concentrations of PMS (1–10 μM, 24 h) displayed pronounced apoptogenicity as detected by annexin V-ITC/propidium iodide flow cytometry, and PMS-induced cell death was suppressed by antioxidant (NAC) or pan-caspase inhibitor (zVAD-fmk) cotreatment. Gene expression array analysis in A375 melanoma cells (PMS, 10 µM; 6 h) revealed transcriptional upregulation of heat shock (*HSPA6*, *HSPA1A*), oxidative (*HMOX1*) and genotoxic (*EGR1*, *GADD45A*) stress responses, confirmed by immunoblot detection demonstrating upregulation of redox regulators (NRF2, HO-1, HSP70) and modulation of pro- (BAX, PUMA) and anti-apoptotic factors (Bcl-2, Mcl-1). PMS-induced oxidative stress and glutathione depletion preceded induction of apoptotic cell death. Furthermore, the mitochondrial origin of PMS-induced superoxide production was substantiated by MitoSOX-Red live cell fluorescence imaging, and PMS-induced mitochondriotoxicity (as evidenced by diminished transmembrane potential and oxygen consumption rate) was observable at early time points. After demonstrating NADPH-driven (SOD-suppressible) superoxide radical anion generation by PMS employing a chemical NBT reduction assay, PMS-induction of oxidative genotoxic stress was substantiated by quantitative Comet analysis that confirmed the introduction of formamido-pyrimidine DNA glycosylase (Fpg)-sensitive oxidative DNA lesions in A375 melanoma cells. Taken together, these data suggest feasibility of repurposing the biochemical reactant PMS as an experimental pro-oxidant targeting mitochondrial integrity and redox homeostasis for the apoptotic elimination of malignant melanoma cells.

## 1. Introduction

Cumulative research suggests a causative involvement of altered redox homeostasis and reactive oxygen species (ROS)-dependent signaling in the control of cancer cell survival, proliferation and invasiveness [1]. It has also been suggested that constitutively elevated levels of oxidative stress and dependence on mitogenic and anti-apoptotic reactive oxygen species (ROS) signaling represent a specific vulnerability of malignant cells that can be selectively targeted by redox-directed chemotherapeutics [1,2,3]. Moreover, it has been shown that mitochondriotoxic molecular interventions impairing structural and functional integrity of mitochondria may contribute to redox-directed elimination of cancer cells [4,5,6]. Thus, small molecule inducers of oxidative stress (i.e., pro-oxidants) are promising experimental therapeutics targeting malignant cells [7].

**Figure 1:**
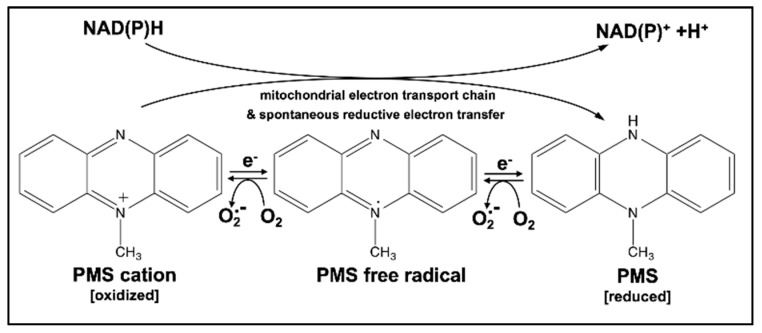
Redox reactivity of Phenazine Methosulfate (PMS). Reductively (NADPH)-driven PMS redox cycling involving single- or two-electron transfer reactions can cause reactive oxygen species (ROS) production with formation of superoxide radical anions. The three redox states of PMS are depicted.

Aiming at the identification of prooxidant pharmacophores with heretofore unexplored potential for experimental redox-directed chemotherapy we first screened a focused library of small molecule biochemical redox probes including photometric tetrazolium dyes and related reactants (nitroblue tetrazolium (NBT), nitroblue formazane (NBF), 3-(4,5-dimethylthiazol-2-yl)-2,5-diphenyltetrazolium bromide (MTT), 3-(4,5-dimethylthiazol-2-yl)-5-(3-carboxymethoxyphenyl)-2-(4-sulfophenyl)-2H-tetrazolium (MTS), other tetrazolium-based reactants (WST-1, WST-8), and non-tetrazolium based reactants including 7-hydroxy-3H-phenoxazin-3-one 10-oxide (resazurin) and phenazine methosulfate (PMS; 5-methylphenazinium methyl sulfate; CAS No. 299-11-6)). Based on the initial observation of promising pro-oxidant and apoptogenic activities, distinguishing PMS from all other compounds contained in our redox panel, we then focused our research efforts on this synthetic N-alkylphenazinium cation-based redox cycler (Figure 1), employed widely as an electron transfer reactant that couples NAD(P)H generation to the reduction of tetrazolium salts (such as MTT) in standard biochemical cell viability assays (allowing for the photometric assessment of mitochondrial respiratory function). Intriguingly, previous research has demonstrated that PMS can serve as an efficient source of superoxide radical anion generation observable in chemical and cellular systems, activities that are based on its ability to serve as a single- or two-electron redox system under physiological conditions (E^0^’ = +0.063 V), transferring electrons from biochemical reducing equivalents (such as NAD(P)H) onto oxygen [8,9]. Moreover, it has been shown that PMS can abstract electrons from the mitochondrial respiratory chain with subsequent transfer to molecular acceptors including oxygen or biochemical probes such as tetrazolium dyes, and PMS redox cycling with superoxide formation has been shown to cause oxidative inactivation of aconitase in cultured mammalian cells [10,11]. Interestingly, phenazine-based redox-active natural products of microbial origin, including pyocyanin (5-Methyl-1(5*H*)-phenazinone) derived from the Gram-negative pathogen pseudomonas aeruginosa, have been identified as redox-based virulence factors involved in quorum sensing and host tissue damage [12,13]. 

Here, we present novel experimental evidence supporting activity of PMS as a prooxidant and mitochondriotoxic redox reactant, suggesting feasibility of repurposing this superoxide generating pharmacophore for the therapeutic apoptogenic elimination of malignant melanoma cells. 

## 2. Results

### 2.1. PMS Induces Caspase-Dependent Death in A375 Malignant Melanoma Cells 

**Figure 2:**
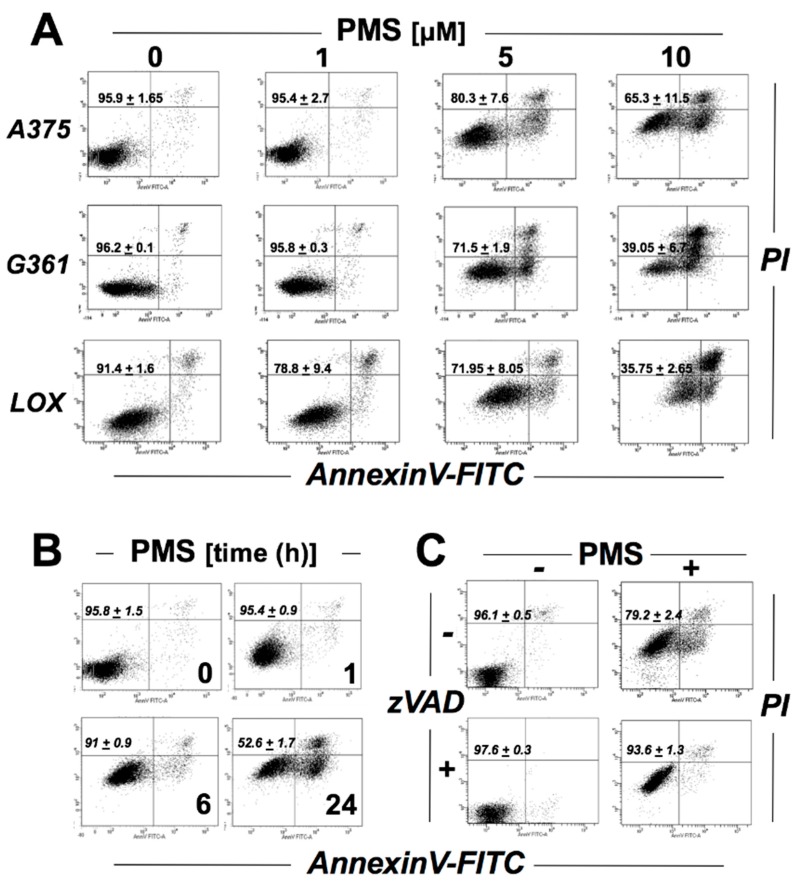
PMS treatment impairs viability of human malignant melanoma cells inducing caspase-dependent cell death. Viability of PMS-exposed cells was monitored using flow cytometric analysis (annexin V-PI staining). Numbers in quadrants indicate viable (AV-negative, PI-negative) in percent of total gated cells (mean ± SD, *n* = 3)]. (**A**) Dose response relationship (PMS 1–10 µM, 24 h) examined in a panel of cultured human malignant melanoma cells (A375, LOX, G361). (**B**) Time course of cytotoxicity examined in PMS-exposed A375 cells (10 µM, 1–24 h). (**C**) PMS-induced (5 µM, 24 h) impairment of A375 cell viability examined in the absence or presence of the pancaspase inhibitor zVAD-fmk (40 µM).

First, to examine the effect of PMS on cell viability, a dose response relationship of PMS-induced cell death was determined using flow cytometric analysis (annexinV-FITC/propidium iodide staining) in a panel of human malignant melanoma cells (A375, G361, LOX). At low micromolar concentrations (5–10 µM; 24 h exposure) PMS caused pronounced induction of cell death observable in all cell lines (Figure 2). Given the established role of A375 cells as a representative model of BRAF^V600E^-driven melanoma, we then focused our more detailed mechanistic studies on this cell line. Time course analysis (Figure 2B; 10 µM PMS) indicated that the full extent of PMS-induced cell death was observable only after prolonged exposure (24 h) not detectable at earlier time points (with cells displaying full viability at 6 h exposure time); a similar time course with attenuated PMS-induced cytotoxicity was detectable at PMS concentrations as low as 5 µM (Figure S1; upper row: 5 µM; lower row: 1 µM). Importantly, pre-treatment with the caspase inhibitor zVAD-fmk (40 µM, 1 h) protected cells from PMS-induced cell death (Figure 2C). Taken together, the characteristics of PMS-induced cell death (i.e., annexin V-positivity (Figure 2A), time-dependence (Figure 2B), and zVADfmk-dependent rescue (Figure 2C)) are consistent with the occurrence of apoptosis.

### 2.2. Array Analysis Reveals Upregulation of Heat Shock, Oxidative, and Genotoxic Stress Response Gene Expression in PMS-Exposed A375 Malignant Melanoma Cells 

**Figure 3:**
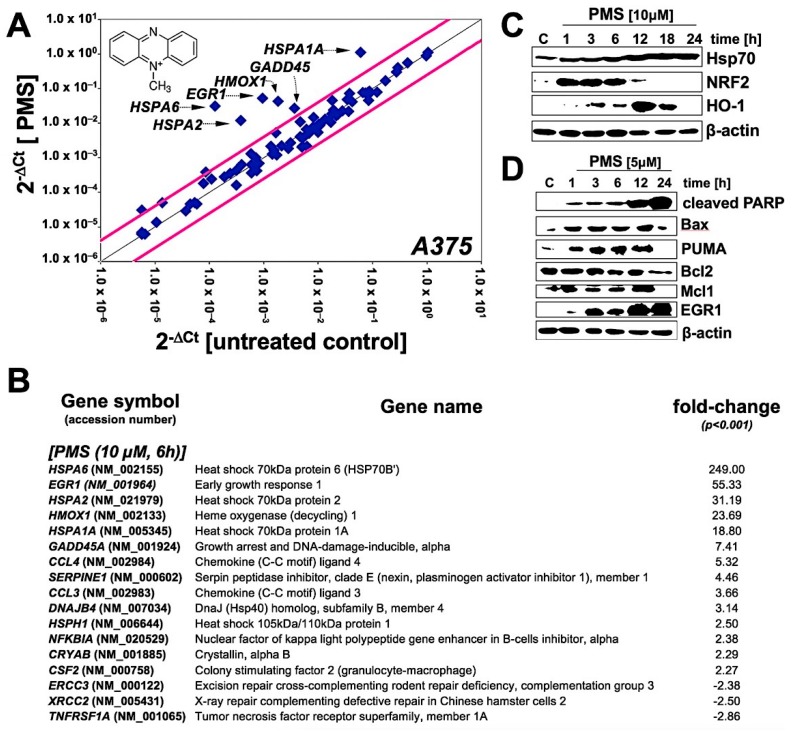
Gene expression array analysis performed in human A375 malignant melanoma cells exposed to PMS. (**A**) The scatter blot depicts differential gene expression as detected by the RT^2^ Human Stress and Toxicity Profiler^TM^ PCR Expression Array technology (PMS: 10 µM, 6 h exposure). Upper and lower lines: cut-off indicating fourfold up- or down-regulated expression, respectively. Arrays were performed in three independent repeats and analyzed using the two-sided Student’s *t* test. (**B**) Numerical expression changes (PMS versus untreated) (*n* = 3, mean + SD; (*p* < 0.001)). (**C**) Modulation of stress response protein levels in PMS-exposed (10 µM, 1–24 h) A375 cells as assessed by immunoblot analysis. (**D**) Modulation of pro- and anti-apoptotic regulator protein levels in PMS-exposed (5 µM, 1–24 h) A375 cells as assessed by immunoblot analysis.

To further characterize the molecular basis underlying PMS-induced melanoma cell death, gene expression array analysis was performed using the RT^2^ Human Stress and Toxicity Profiler^TM^ PCR Expression Array technology (Figure 3). To this end, A375 cells were exposed to a cytotoxic concentration of PMS (10 µM) and analyzed at a time point at which viability was still maintained (6 h; Figure 2B). Out of 84 stress-response genes profiled on the array, 17 displayed pronounced expression changes (by up to almost 250-fold), indicative of proteotoxic (heat shock), oxidative and genotoxic stress (Figure 3A,B): A broad PMS-induced cellular heat shock response was substantiated by pronounced overexpression of *Heat shock 70kDa protein 6* (encoded by *HSPA6*; 249-fold), *Heat shock 70kDa protein 2* (encoded by *HSPA2*; 31-fold), *Heat shock 70kDa protein 1A* (encoded by *HSPA1A*; 19-fold), *DnaJ (Hsp40) homolog, subfamily B, member 4* (encoded by *DNAJB4*; 3-fold), *Heat shock 105kDa/110kDa protein 1* (encoded by *HSPH1*; 3-fold), and *Crystallin, alpha B* (encoded by *CRYAB*; 2-fold). Likewise, upregulation of the cellular antioxidant enzyme *heme oxygenase-1* (encoded by *HMOX1*; 24-fold) was observed. In addition, expression of genotoxic stress sensor and response factors including *Growth arrest and DNA-damage-inducible, alpha* (encoded by *GADD45A*; 7-fold) and the stress-responsive transcription factor and tumor suppressor *Early growth response protein 1* (also called Zif268 (zinc finger protein 225) encoded by *EGR1*; 55-fold) was induced; interestingly, expression of DNA repair enzyme encoding genes including *Xeroderma pigmentosum group B complementing* (*ERCC3*; 2-fold) and *X-ray repair complementing defective repair in Chinese hamster cells 2* (*XRCC2*; 3-fold) was downregulated in response to PMS treatment. 

Follow up experimentation employing immunoblot analysis revealed PMS-induced expression changes detectable at the protein level that were consistent with those observed at the mRNA level (Figure 3C,D): Expression of heat shock and oxidative stress response proteins such as Hsp70, the antioxidant response regulatory transcription factor NRF2, and HO-1 was upregulated in response to PMS (Figure 3C). Importantly, expression of numerous apoptotic regulators was modulated favoring initiation and execution of apoptosis, including upregulation of pro-apoptotic factors (i.e., Bax, PUMA, cleaved PARP1, EGR1), accompanied by downregulation of anti-apoptotic factors (Mcl-1, Bcl-2; Figure 3D). 

### 2.3. PMS Induces Mitochondriotoxicity in Human A375 Malignant Melanoma Cells Characterized by Ultrastructural Changes, Mitochondrial Respiratory Impairment, Loss of Transmembrane Potential, and Superoxide Production 

**Figure 4:**
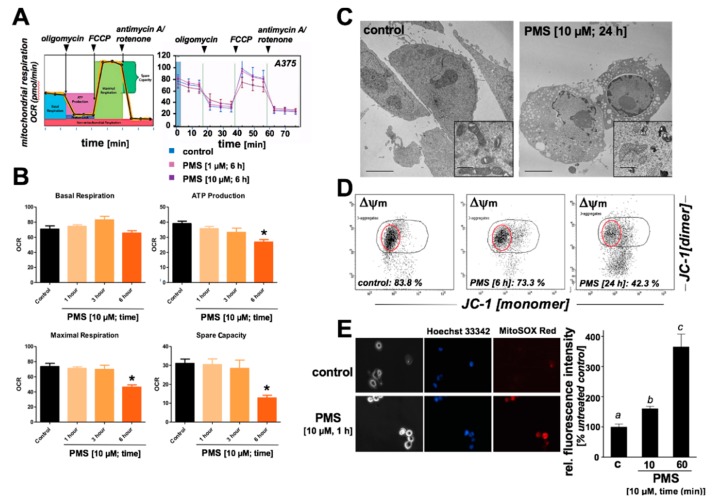
PMS exposure impairs mitochondrial oxygen consumption rate (OCR) and causes loss of mitochondrial transmembrane potential (Δψm) with superoxide generation in human A375 malignant melanoma cells. A375 melanoma cells were exposed to PMS, and effects on mitochondrial function were assessed. (**A**) Mitochondrial OCR was monitored using a Seahorse Extracellular Flux (XF) 96 analyzer in real-time in live A375 cells exposed to PMS (1–6 h preincubation time; 1–10 µM); left panel: general metabolic parameters as assessed by Extracellular Flux analysis; right panel: OCR as a function of PMS preincubation and addition of oligomycin, FCCP, and antimycin A/rotenone (*n* = 3). (**B**) Bar graphs depict PMS (10 µM, 1–6 h) effects on basal respiration, ATP-linked OCR, maximal OCR, and reserve capacity (*n* = 3; * p < 0.05). (**C**) PMS-induced ultrastructural changes in A375 cells observed by transmission electron microscopy (PMS 10 µM, 24 h; TEM × 2650; scale bar: 2 µm). Inserts display mitochondria with alterations in PMS-treated cells (TEM × 20,500; scale bar: 500 nm). (**D**) Mitochondrial transmembrane potential was determined using flow cytometric analysis of JC-1 stained cells (10 µM PMS, ≤24 h). Numbers indicate cells displaying fully functional/polarized mitochondrial potential (marked by red circle) in percent of total cells; a representative experiment out of three similar repeats is displayed. (**E**) Time course of mitochondrial superoxide production in response to PMS (10 μM) as detected by MitoSOX Red^TM^ fluorescence microscopy (including Hoechst 33342-based nuclear counterstaining; 40 fold magnification). Bar graph displays quantitative analysis of relative fluorescence intensity (% untreated control; *n* = 3).

Next, based on prior published evidence that PMS causes redox interference with the mitochondrial electron transport chain, effects of PMS exposure on mitochondrial function were examined in A375 melanoma cells (Figure 4). First, mitochondrial oxygen consumption rate (OCR) was monitored using a Seahorse Extracellular Flux (XF) 96 analyzer in real-time in live A375 cells exposed to PMS (1–6 h preincubation time; 1–10 µM; Figure 4A,B). When OCR was determined as a function of PMS preincubation and addition of established respiratory co-modulators (oligomycin, FCCP, antimycin A/rotenone), it was observed that PMS (10 µM, 6 h) treatment was associated with significant (*p* < 0.05) inhibition of ATP-linked OCR (approximately 25% reduction), maximal respiration (approximately 40% reduction), and spare capacity (approximately 60% reduction), whereas basal respiration remained unaffected. Further evidence in support of mitochondriotoxic effects of PMS treatment was provided by the observation that at later time points (24 h) caused pronounced ultrastructural changes detectable by electron microscopy, characterized by extensive nuclear condensation, cytosolic vacuolization, and mitochondrial swelling with cristae abnormalities and fragmentation (Figure 4C). Likewise, a significant impairment of mitochondrial transmembrane potential (Δψm) as determined by flow cytometric analysis of JC-1 stained cells (10 µM PMS) was already observable upon 6 h continuous PMS exposure (Figure 4D), and more than 55% of PMS treated cells displayed loss of transmembrane potential at a later time point (24 h). Consistent with a PMS-induced disruption of functional respiratory electron transport, a more than three-fold upregulation of mitochondrial superoxide levels was already detectable at early time points of PMS exposure (1 h) as assessed by detection of increased MitoSOX Red relative fluorescence intensity (Figure 4E), an effect preceding functional impairment (Figure 4A,B), ultrastructural changes (Figure 4C), and loss of transmembrane potential (Figure 4D). 

### 2.4. PMS Induces Oxidative Stress in Human A375 Malignant Melanoma Cells Associated with Glutathione Depletion And Impairment Of Genomic Integrity 

**Figure 5:**
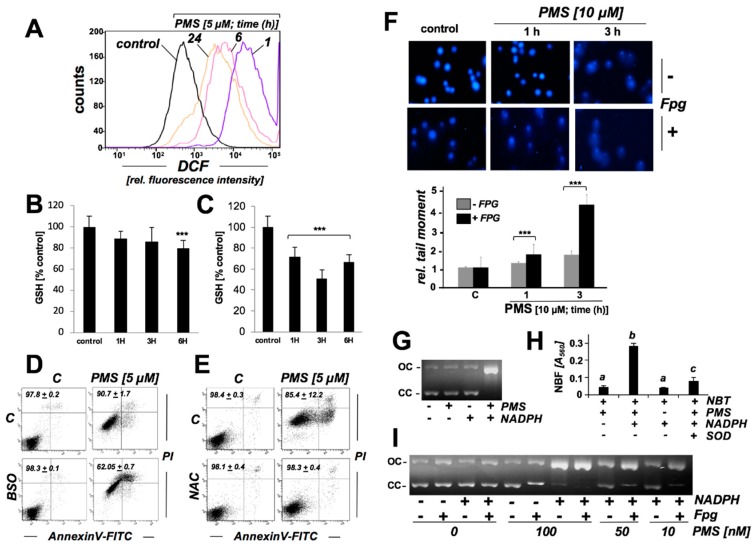
PMS exposure induces redox dysregulation with glutathione depletion and oxidative impairment of genomic integrity in human A375 malignant melanoma cells, a genotoxic effect that can also be observed in an in vitro plasmid cleavage assay. (**A**) Analysis of oxidative stress in PMS-exposed A375 cells (10 µM, ≤ 24 h) as monitored by flow cytometric detection of DCF fluorescence. One set of histograms representative of three repeats is shown. (**B**,**C**) Time course (≤ 6 h) of PMS-modulation of intracellular reduced glutathione content in A375 cells (panel **B**: 1 µM; panel **C**: 10 µM), normalized to cell number (mean ± SD, *n* ≥ 3; *** *p* < 0.001). (**D**,**E**) After preincubation (24 h) with BSO (1 mM, panel **C**) or NAC (10 mM, panel **D**), PMS-induced (5 µM, 24 h) A375 cell death was assessed by flow cytometric analysis of AV/PI-stained cells (mean ± SD, *n* ≥ 3). (**F**) Fpg-enhanced Comet assay assessing cellular genotoxic stress (40 fold magnification; PMS: 10 µM; (analysis post treatment: ≤ 3 h); bar graph: tail moment analysis (*n* ≥ 100 per group; mean ± S.E.M; *** *p* < 0.001). (**G**) PMS-induced ΦX174-plasmid cleavage in vitro (*OC*: open circular; *CC* closed circular; PMS: 1 µM; NADPH: 200 µM; 3 h reaction time). (**H**) PMS-induced superoxide production as assessed by NBT reduction performed in the presence or absence of SOD (3000 μ/mL; *n* = 3). NBF was detected photometrically (A_560_ nm). (**I**) PMS dose response of Fpg-enhancement of ΦX174-plasmid cleavage in vitro (*OC*: open circular; *CC* closed circular; PMS: ≤ 100 nM; NADPH: 200 µM; 3 h reaction time), indicative of oxidative DNA lesions induced by NADPH/PMS exposure.

Next, PMS-induced occurrence of oxidative stress in A375 melanoma cells was explored in further detail utilizing the intracellular redox probe DCFH-DA (Figure 5A). To this end, cells were exposed to PMS (10 µM) for up to 24 h, followed by DCFH-DA loading and flow cytometric analysis of DCF fluorescence intensity indicative of oxidative stress. A short PMS exposure time (1 h) was sufficient to induce an approximately fifty-fold increase in DCF fluorescence intensity, and extended PMS exposure (6–24 h) caused a pronounced yet somewhat diminished increase in DCF fluorescence intensity (approximately 10-fold), an observation consistent with a partial recovery from PMS-induced oxidative stress, possibly due to upregulation of antioxidant responses in cells that maintain viability up to 24 h PMS exposure time (Figure 2B and Figure 3A–C). 

Next, PMS-induced depletion of reduced glutathione (GSH) was monitored (Figure 4B,C). Significant reduction by almost 30% was detectable within 1 h exposure time, when A375 cells were exposed to a cytotoxic (cell death-inducing) concentration of PMS (10 µM; Figure 5C). Upon extended exposure (3 h), GSH levels were reduced even further (by almost 50%). At 6 h exposure time GSH levels were increased to those levels observed after 1 h exposure, an observation consistent with a partial attenuation of cellular PMS-induced oxidative stress as observed with DCF (Figure 5A). Even at lower concentrations of PMS (1 µM; a concentration not associated with the induction of A375 cell death; Figure 2A), a significant reduction in GSH levels by approximately 20% occurred within 6 h exposure time (Figure 5B). Consistent with a mechanistic involvement of oxidative stress and GSH depletion in PMS-induced cell death, pronounced potentiation of PMS-cytotoxicity was observed in cells exposed to PMS (5 µM, 24 h) after pharmacological glutathione depletion (using buthionine sulfoximine (BSO), 1 mM; 24 h pretreatment; Figure 5D). Vice versa, NAC preincubation (N-acetyl-L-cysteine; 10 mM, 24 h), an established GSH biosynthesis precursor, protected cells against PMS-induced cytotoxicity (Figure 5E).

After exploring the molecular nature of PMS-induced redox dysregulation, we examined the possibility that PMS-induced cellular oxidative stress may be associated with the generation of genomic DNA damage. Indeed, expression array analysis had indicated the upregulation of *GADD45A, EGR1,* and a number of DNA repair genes (*ERCC3*, *XRCC2*), suggesting induction of genotoxic stress as a result of PMS exposure (Figure 3). Likewise, upregulation of *EGR1* expression at the protein level was observable within 1 h of PMS exposure (Figure 3D). First, the occurrence of PMS-induced impairment of genomic integrity and induction of oxidative DNA damage was assessed in A375 cells using alkaline single cell gel electrophoresis performed with or without formamido-pyrimidine DNA-glycosylase (Fpg)-digestion (Fpg-modified Comet assay; Figure 5F) [14,15]. Indeed, PMS treatment (10 μM) caused a pronounced increase in average comet tail moment (approximately 4-fold over untreated control; 3 h exposure time). Strikingly, this increase in average tail moment was observable only upon Fpg-digestion, an effect consistent with the introduction of oxidative base lesions by PMS followed by Fpg-dependent excision of formamidopyrimidine and 8-oxodG residues and strand breaks at apurinic/apyrimidinic sites. Using this method, PMS-induced oxidative DNA damage was detectable as early as within 1 h exposure time causing an approximately 30% increase in average comet tail moment.

Next, in order to gain further mechanistic insights and to examine PMS-induced chemical oxidative DNA damage (that might occur independent of mitochondrial involvement), impairment of genomic integrity was investigated in a cell-free plasmid cleavage assay (Figure 5G–I). This assay detects treatment-induced single strand breaks through the formation of open circular (OC; ’nicked’) plasmid forms, characterized by reduced electrophoretic mobility as compared to the more compact (undamaged) closed circular (CC) form [16]. Strikingly, PMS-induced strand breaks only occurred in the presence of a reducing agent (NADPH) included in the reaction mixture (Figure 5G). Moreover, when the dose-response relationship of PMS-induced plasmid cleavage was examined, PMS-induced (NADPH-dependent) cleavage occurred irrespective of Fpg-digestion, observable at PMS concentrations as low as 100 nM (Figure 5I). However, at lower PMS concentrations (10–50 nM) plasmid cleavage was strongly enhanced by Fpg-digestion. These observations are consistent with reductively-driven (i.e., NADPH-dependent) PMS-induced oxidative cleavage, causing complete plasmid cleavage at high PMS concentrations (≥100 nM; irrespective of Fpg treatment), whereas at low PMS concentrations (10–50 nM) oxidative cleavage is attenuated and requires Fpg-enhancement for detection. Next, PMS-driven superoxide formation was confirmed in a chemical/acellular system employing the photometric NBT-reduction assay performed in the absence or presence of erythrocyte-derived superoxide dismutase (SOD; Figure 5H). As already suggested by the observations on NADPH-driven PMS-dependent plasmid cleavage, (Figure 5G–I), it was observed that PMS-dependent NBT reduction required the reductive driving force of NADPH resulting in stochiometric nitroblue formazan (NBF) formation; consequently, inclusion of SOD, and enzyme detoxifying/converting superoxide radical anions from the reaction mixture, was able to significantly antagonize PMS/NADPH-driven NBT reduction and NBF formation (Figure 5H). Taken together, these data suggest that oxidative modification of plasmid DNA and Fpg-dependent cleavage can occur downstream of spontaneous (chemical), reductively-driven PMS-redox cycling and that PMS can cause oxidative genomic DNA damage observable in malignant melanoma cells.

### 2.5. PMS Preferentially Induces Cell Death in Vemurafenib-Resistant A375 Melanoma Cells (A375R)

**Figure 6:**
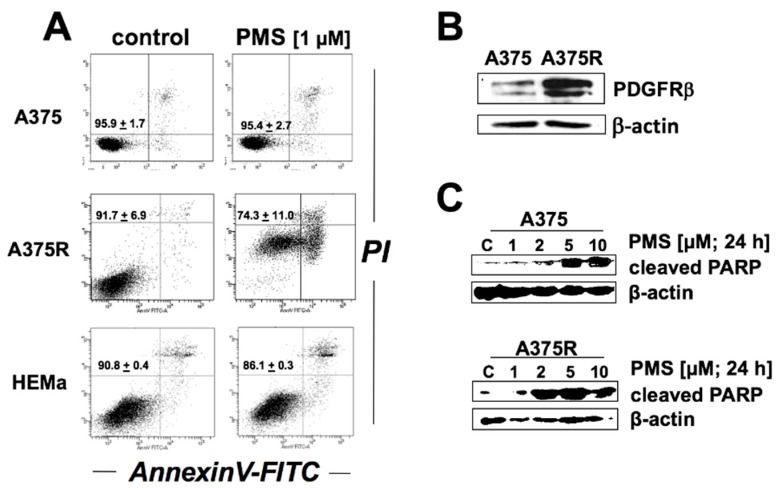
PMS treatment preferentially impairs viability of vemurafenib-resistant human malignant melanoma cells with induction of caspase-dependent PARP-cleavage. (**A**) Differential cytotoxicity of PMS (1 µM; 24 h) was examined in a focused panel of cultured human malignant melanoma cells (A375, A375R (vemurafenib-resistant A375)) and primary melanocytes (HEMa) using flow cytometric analysis (annexin V-PI staining). Numbers in quadrants indicate viable (AV-negative, PI-negative) in percent of total gated cells (mean ± SD, *n* = 3). (**B**) Immunoblot-detection of PDGFRβ-upregulation in A375R cells, an established signature molecular change characteristic of vemurafenib-resistant melanoma cells. (**C**) PMS-induced (1–10 µM, 24 h) PARP1-cleavage, comparing A375 versus A375R malignant melanoma cells.

Previous research has indicated therapeutic efficacy of mitochondriotoxic agents as experimental cancer chemotherapeutics, and vemurafenib-resistant melanoma cells have been demonstrated to display a specific vulnerability to mitochondriotoxic agents, a sensitivity attributable to metabolic rewiring and dependency of vemurafenib-resistant cells on mitochondrial energy production [4,5,6,17,18]. Thus, we compared the apoptogenicity of PMS in a focused cell panel containing healthy primary human melanocytes (HEMa) and vemurafenib-resistant (A375R) versus vemurafenib-sensitive A375 melanoma cells (Figure 6). A375R cells were generated by continuous selective culture in vemurafenib; immunoblot-detection confirmed PDGFRβ-upregulation in these cells, an established signature molecular change characteristic of vemurafenib-resistant melanoma cells (Figure 6B) [19]. 

Indeed, flow cytometric analysis revealed preferential cytotoxicity targeting A375R cells, an effect observable at a low PMS concentration (1 µM; 24 h) that does not inactivate A375 wildtype cells or primary human melanocytes (Figure 6A). Likewise, it was observed that PMS treatment (1–10 µM, 24 h) induces pronounced PARP1-cleavage in A375R malignant melanoma cells even at low concentrations that do not cause this effect in A375 cells. In addition, preliminary flow cytometric viability analysis assessing the differential cytotoxicity of PMS, comparing sensitivity of malignant and non-transformed cultured human skin cells, revealed that Hs27 human diploid dermal fibroblasts maintained full viability at PMS concentrations (5–10 µM; 24 continuous exposure; that are effective in eliminating malignant melanoma cells contained in our test panel (A375, A375R, G361, LOX; Figure 2 and Figure 6), an observation suggesting a potential therapeutic window associated with PMS-apoptogenicity, awaiting further elucidation in future follow-up studies.

Taken together, these prototype data suggest that PMS treatment preferentially impairs viability of vemurafenib-resistant human malignant melanoma cells with induction of caspase-dependent PARP1-cleavage, an observation consistent with the reported hypersensitivity of vemurafenib-resistant melanoma cells to mitochondriotoxic therapeutic intervention.

## 3. Discussion

Cumulative experimental evidence suggests that altered redox metabolism is involved in the control of tumorigenesis, and redox-directed interventions including the pharmacological induction of redox dysregulation and oxidative stress are rapidly emerging as promising strategies targeting cancer cells [1,2,3,20,21,22]. 

Various classes of therapeutic pro-oxidants (e.g., organic endoperoxides and Michael-type electrophiles, antagonists of glutathione and thioredoxin metabolism, MnSOD-mimetics, Nrf2 antagonists, and inducers of ferroptosis) have been examined as promising experimental cancer therapeutics [7,20,23,24,25,26]. It has also been suggested that oncogene-driven proliferative signaling and dependence on ROS-driven mitogenic redox dysregulation may represent a molecular vulnerability of malignant cells that can be targeted by specific redox chemotherapeutics (21,22,25). Among small molecule prooxidants, catalytic redox cyclers, facilitating electron transfer from cellular reducing agents (such as NAD(P)H) onto molecular oxygen, have attracted considerable attention, and prototype experiments have explored redox cycler-dependent production of apoptogenic ROS (including superoxide and hydrogen peroxide) in cancer cells [3,25,27,28]. However, identification and preclinical development of versatile redox cycler pharmacophores amenable to further therapeutic development remains of much interest.

Here, we report for the first time that the biochemical redox reactant PMS may serve as a novel experimental chemotherapeutic that can induce apoptosis in human malignant melanoma cells. In a panel of malignant human melanoma cells low micromolar concentrations of PMS (≤ 10 μM, 24 h) displayed pronounced apoptogenicity as detected by annexin V-FITC/propidium iodide flow cytometry and immunodetection of PARP-1 cleavage; PMS-induced cell death was suppressed by antioxidant or pan-caspase inhibitor co-treatment (Figure 2 and Figure 6). Gene expression array analysis in A375 melanoma cells (PMS, 10 µM; 6 h) revealed transcriptional upregulation of heat shock (*HSPA6*, *HSPA1A*), oxidative (*HMOX1*), and genotoxic (*EGR1*, *GADD45A*) stress responses, confirmed by immunoblot detection that included redox regulators (NRF2, HO-1, EGR1, HSP70) and modulation of pro-apoptotic (BAX, PUMA) as well as anti-apoptotic factors (Bcl-2, Mcl-1) (Figure 3). PMS-induced oxidative stress and glutathione depletion were detectable at early time points (1–3 h) that preceded induction of apoptotic cell death (Figure 4 and Figure 5). Furthermore, the mitochondrial origin of PMS-induced superoxide production was substantiated by MitoSOX-Red live cell fluorescence imaging, and PMS-induced impairment of mitochondrial function (including diminished transmembrane potential and oxygen consumption rate) was observable at early time points (Figure 4). NADPH-driven chemical superoxide generation by PMS was demonstrated examining SOD-suppressible NBT reduction (Figure 5). Likewise, PMS-induction of oxidative genotoxic stress was then substantiated demonstrating plasmid cleavage in a chemical assay and by quantitative Comet analysis that indicated the introduction of formamido-pyrimidine DNA glycosylase (Fpg)-sensitive DNA lesions (Figure 5). 

Melanoma, a malignant tumor derived from melanocytes, causes the majority of deaths attributed to skin cancer. Despite recent progress in the design of melanoma-targeted therapies such as the ^V600E^-mutation-directed BRAF-inhibitor vemurafenib [29,30], efficacy of chemotherapeutic intervention directed against the metastatic stage of the disease remains limited due to rapid emergence of resistance creating an urgent need for the identification and development of improved antimelanoma agents [31,32]. Dysregulation of oxidative stress has been observed in human melanoma tissue contributing to the notorious chemoresistance of metastatic melanoma cells, and redox dysregulation and reprogramming of mitochondrial energy metabolism have been identified as key mechanisms underlying BRAF^V600^-melanoma resistance to targeted kinase inhibitors [5,17,33,34,35]. Indeed, experimental efficacy of mitochondriotoxic therapeutics that target mitochondrial dependency of vemurafenib-resistant melanoma cells has been substantiated [5]. Remarkably, our prototype experiments indicate that vemurafenib-resistant A375 malignant cells display higher sensitivity to PMS treatment (Figure 6), an observation that might suggest feasibility of overcoming vemurafenib-resistance through induction of PMS-mediated mitochondriotoxicity and oxidative stress. 

Taken together these data indicate that the biochemical electron transfer reactant PMS is a pro-oxidant that can be repurposed for the apoptotic elimination of malignant melanoma cells through induction of lethal oxidative and mitochondriotoxic stress. However, it remains to be seen if reductively-driven ROS formation by PMS redox cycling as envisioned in the prototype experiments presented here can be used for targeted molecular redox interventions. For example, achieving a therapeutic window of sufficient width that eliminates malignant cells while sparing healthy tissue might require a conjugation strategy, coupling this redox pharmacophore as a cytotoxic warhead and pro-oxidant payload in antibody drug conjugates (ADCs), a targeted delivery approach that has been successfully pursued with pyrrolobenzodiazepine- and auristatin-ADCs, inactivating tumors cells in human patients with high selectivity. Moreover, given the chemical versatility and accessibility of N-methylated phenazinium cations it seems feasible to generate redox-inactive prodrugs that depend on metabolic intracellular conversion to PMS for activation of this cytotoxic redox cycler.

## 4. Materials and Methods 

### 4.1. Chemicals

All chemicals were purchased from Sigma Chemical Co (St. Louis, MO, USA). 

### 4.2. Cell Culture

Malignant human melanoma cells (A375, LOX-IMVI, G-361) from ATCC (Manassas, VA, USA) were cultured in RPMI medium (10% FBS and 2 mM L-glutamine) or McCoy’s 5a medium (10% FBS), respectively. A375 cells resistant to the BRAF^V600E^-kinase inhibitor vemurafenib (A375R) were generated by continuous selective culture (>12 weeks) employing increasing concentrations of vemurafenib (0.1–5 µM) with subsequent maintenance culture (5 µM) following a published standard procedure [36,37]. Primary human epidermal melanocytes (adult skin, lightly pigmented: HEMa-LP from Life Technologies, Grand Island, NY, USA; abbreviated HEMa) were cultured using Medium 154 medium supplemented with HMGS-2 growth supplement. HEMa cells were passaged using recombinant trypsin/EDTA and defined trypsin inhibitor. Human diploid dermal fibroblasts (Hs27, ATCC) were cultured as described by us before [14]. Cells were maintained at 37 °C in 5% CO_2_, 95% air in a humidified incubator. 

### 4.3. Flow Cytometry Analysis of Cell Viability 

Viability and induction of cell death (early and late apoptosis/necrosis) were examined by annexinV-FITC/propidium iodide (PI) dual staining of cells followed by flow cytometric analysis [38]. Cells (100,000) were seeded on 35 mm dishes and received drug treatment at different concentrations 24 h later. Cells were harvested 24 h after treatment and cell staining was performed using an apoptosis detection kit according to the manufacturer’s specifications (Sigma Chemical Co, APOAF). Viable cells are located in the bottom left quadrant (annexinV-FITC^−^/PI^−^), whereas early apoptotic and late apoptotic/necrotic cells are located in the bottom right (annexinV-FITC^+^/PI^−^) and top right quadrants (annexinV-FITC^+^/PI^+^), respectively.

### 4.4. Human Stress and Toxicity Pathfinder RT^2^Profiler^TM^ PCR Expression Array

After being exposed to 10 µM PMS for 6 hours, total cellular RNA (3 × 10^6^ cells) was prepared according to a standard procedure using the RNeasy kit (Qian, Valencia, CA, USA). Reverse transcription was performed using the RT^2^ First Strand kit (Super Array, Frederick, MD, USA) and 5 mg total RNA. The RT^2^ Human Stress and Toxicity Pathfinders PCR Expression Array (Super Array) profiling the expression of 84 stress-related genes was run using the following PCR conditions: 95 °C for 10 min, followed by 40 cycles of 95 °C for 15 s alternating with 60 °C for 1 min (Applied Biosystems 7000 SDS, Carlsbad, CA, USA). Gene-specific product was normalized to GAPDH and quantified using the comparative (ΔΔCt) Ct method as described in the ABI Prism 7000 sequence detection system user guide as published earlier [38]. Expression values were averaged across three independent array experiments, and standard deviation was calculated for graphing and statistical analysis as published before. 

### 4.5. Immunoblot Detection 

After cellular protein extraction, proteins were separated using SDS-PAGE (12% gel), transferred to nitrocellulose membrane, and developed. The following antibodies were used: anti-Hsp70 mouse monoclonal (C92F3A-5; Enzo Life Sciences, Farmingdale, NY, USA), anti-NRF2 rabbit polyclonal (13032; Santa Cruz, Dallas, TX, USA), anti-NQO1 mouse monoclonal (28947; abcam, Cambridge, UK), anti-HO-1 rabbit monoclonal (5853; Cell Signaling Technology, Danvers, MA, USA), anti-cleaved-PARP rabbit polyclonal (9541; Cell Signaling Technology), anti-Bax rabbit polyclonal (2772; Cell Signaling Technology), anti-Mcl 1 rabbit polyclonal (4572; Cell Signaling Technology), anti-Bcl2 rabbit monoclonal (12450; Cell Signaling Technology), anti-PUMA rabbit monoclonal (12450; Cell Signaling Technology), anti-EGR1 rabbit monoclonal (4154; Cell Signaling Technology), anti-PDGF receptor beta (28E1) rabbit monoclonal antibody (3169; Cell Signaling Technology). The following secondary antibodies were used: HRP-conjugated goat anti-rabbit antibody or HRP-conjugated goat anti-mouse antibody (Jackson ImmunoResearch Laboratories, WestGrove, PA, USA). Equal protein loading was examined by β-actin-detection using a mouse anti-actin monoclonal antibody (Sigma Chemical Co).

### 4.6. PMS-Modulation of Mitochondrial Respiration in a375 Melanoma Cells 

A Seahorse Extracellular Flux (XF) 96 Analyzer (Seahorse Bioscience, Inc, North Billerica, MA, USA) using a specialized oxygen consumption rate (OCR) kit (Seahorse XFe96 FluxPaks^TM^, Agilent, Santa Clara, CA, USA) was employed in 96 well format according to the manufacturer’s specifications measuring OCR as a quantitative indicator of mitochondrial respiration as published previously [39,40]. Briefly, in A375 cells exposed to PMS (1–10 µM, ≤ 6 h exposure time), OCR was monitored before and after addition of respiratory inhibitors as follows: First, baseline OCR was established in the absence of modulators. Oligomycin (complex V-inhibitor; 1 µM) was used to establish ATP-linked respiration. The uncoupling protonophore carbonyl cyanide-p-trifluoromethoxyphenylhydrazon (FCCP; 0.3 µM) was used to establish maximal respiratory capacity (established by subtracting nonmitochondrial respiration from FCCP rate). Use of rotenone (1 mM)/antimycin A (0.3 µM; complex I/III inhibitor, respectively) determines non-mitochondrial respiration. Mitochondrial reserve capacity was calculated by subtracting basal respiration from maximal respiratory capacity. Experiments (three independent repeats) were analyzed using the paired Student’s *t*-test. 

### 4.7. Detection of Intracellular Oxidative Stress by Flow Cytometric Analysis 

Induction of intracellular oxidative stress by PMS was analyzed by flow cytometry using 2’,7’-dichlorodihydrofluorescein diacetate (DCFH-DA) as a sensitive non-fluorescent precursor dye according to a published standard procedure [7,38]. Cells were treated with PMS (10 µM, 1–24 h), followed by DCFH-DA loading. Cells were incubated for 60 minutes in the dark (37 °C, 5% CO_2_) with culture medium containing DCFH-DA (5 µg/mL). Cells were then harvested and analyzed immediately by flow cytometry.

### 4.8. MitoSOX Fluorescence Imaging

The production of mitochondrial superoxide was monitored by fluorescence microscopy using the mitochondria-directed superoxide probe MitoSOX Red^TM^ (ThermoFisher, Waltham, MA, USA) according to the manufacturer’s protocol [41]. After PMS exposure, cells were kept in fresh medium containing MitoSOX Red^TM^ (5 µM) in combination with Hoechst 33,342 (ThermoFisher) as a nuclear counterstain for normalization. Cells were washed with DPBS followed by visualization using an EVOS FL Auto Cell Fluorescence Imaging system using the DAPI/RFP light cube. MitoSOX RedTM fluorescence intensity was then quantified using ImageJ software (LOCl, Madison, WI, USA). 

### 4.9. Mitochondrial Transmembrane Potential

Mitochondrial transmembrane potential (Δψm) was assessed using the potentiometric dye JC-1 (5,5′,6,6′-tetrachloro-1,1′,3,3′-tetraethylbenzimidazolyl-carbocyanine iodide; Sigma Chemical Co) as published earlier [41]. Cells were resuspended in PBS containing JC-1 (5 μg/mL, 15 min, 37 °C, 5% CO_2_). Bivariate analysis was performed by flow cytometry (excitation at 488 nm); mitochondrial function was assessed as JC-1 green (depolarized mitochondria, detector FL-1) or red (polarized mitochondria, detector FL-2) fluorescence.

### 4.10. Determination of Reduced Cellular Glutathione Content

Intracellular reduced glutathione was measured using the GSH-Glo Glutathione assay kit (Promega; San Luis Obispo, CA) as described recently [7]. Cells were seeded at 100,000 cells/dish on 35 mm dishes. After 24 h, cells were treated with test compound. At selected time points, cells were harvested by trypsinization and then counted using a Coulter counter. Cells were washed in PBS, and 10,000 cells/well (50 µL) were transferred onto a 96-well plate. GSH-Glo reagent (50 µL) containing luciferin-NT and glutathione-S-transferase was then added followed by 30 min incubation. After addition of luciferin detection reagent to each well (100 µL) and 15 min incubation luminescence reading was performed using a BioTek Synergy 2 Reader (BioTek, Winooski, VT, USA). Data are normalized to GSH content in untreated cells and expressed as means ± SD (*n* = 3).

### 4.11. Transmission Electron Microscopy 

Cells were fixed in situ with 2.5% glutaraldehyde in 0.1 M cacodylate buffer (pH 7.4), post-fixed in 1% osmium tetroxide in cacodyl ate buffer, washed, scraped and pelleted as described recently [42]. Cells were then stained in 2% aqueous uranyl acetate, dehydrated through a graded series (50, 70, 90, and 100%) of ethanol and infiltrated with Spur’s resin (Sigma Chemical Co, EM0300), then allowed to polymerize overnight at 60 °C. Sections (50 nm) were cut, mounted onto uncoated 150-mesh copper grids, and stained with 2% lead citrate. Sections were examined in a CM12 transmission electron microscope (FEI) operated at 80 kV with digital image collection.

### 4.12. Comet Assay (Alkaline Single-Cell Gel Electrophoresis)

The alkaline comet assay was performed according to the manufacturer’s instructions (Trevigen, Gaithersburg, MD, USA) as published recently [14,15]. Cells were then stained with DAPI and imaged with a fluorescence microscope followed by analysis using ImageJ software. At least 100 tail moments for each group were analyzed to calculate the mean ± SD for each group. The Fpg-FLARE assay for assessment of Fpg-induced strand cleavage at oxidized purine bases was performed according to the manufacturer’s instructions (Trevigen) [15].

### 4.13. Plasmid Cleavage Assay

DNA strand breakage was measured by the conversion of supercoiled ΦX-174 RF1 double-stranded DNA (SC-DNA) (New England Biolabs, Ipswich, MA, USA) to open circular form (OC) as described [14,16]. Briefly, reaction combinations (ΦX-174 target DNA (0.6 µg), NADPH (200 µM), PMS (10–500 nM), and SOD (400 u) in PBS) were incubated in the dark (30 µL total volume; 3 h, room temperature), followed by SC/OC separation using standard 1.5% agarose gel electrophoresis [TBE (45 mM tris-borate, 1 mM EDTA, pH 8) with ethidium bromide; 150 V, 90 min]. Optionally, before electrophoresis, for visualization of oxidative DNA damage, the reaction mixture was subjected to Fpg-digestion using a commercial kit (Trevigen).

### 4.14. NBT Assay for PMS-Dependent Superoxide Formation 

Chemical formation of superoxide radical anion in the presence or absence of PMS and NADH was determined using the photometric NBT reduction assay, confirmed by scavenging of superoxide using superoxide dismutase (SOD, from bovine erythrocytes) as reported [16]. In brief, 2 µL of NBT [50 mg/mL (50% EtOH)], 10 µL of PMS (10 mM), and 5 µL of NADPH (disodium salt; 4.7 mM) in the presence or absence of 20 µL of SOD (1135 u) were used, and H_2_O was added (222 µL total volume). After 5 min at room temperature, 100 µL of each sample was then added to a 96-well plate, and the reduction product NBF (nitro-blue diformazan) was detected photometrically at 560 nm. 

### 4.15. Statistical Analysis

Unless indicated differently, the results are presented as mean ± S.D. of at least three independent experiments. Paired Student’s *t*-tests were used to compare the means of groups. A *p* < 0.05 was deemed significant. Where indicated select data were analyzed employing one-way analysis of variance (*ANOVA)* with Tukey’s post hoc test using the Prism 4.0 software (Graphpad, San Diego, CA, USA); means without a common letter differ (*p* < 0.05).

## 5. Conclusions

Our cell culture- and chemical reaction system-based prototype data suggest feasibility of repurposing the electron transfer reactant phenazine methosulfate (PMS) for the apoptotic elimination of malignant melanoma cells through induction of lethal oxidative and mitochondriotoxic stress. Future studies will examine avenues of redox-directed interventions harnessing PMS-dependent induction of cellular oxidative stress that might target a wide array of cancer cells beyond malignant melanoma. Ultimately, the potential for further preclinical development and translation of our prototype experiments will depend on the success of designing improved molecular strategies that would allow the use of PMS and related pharmacophores with an acceptable therapeutic window and target selectivity, enabling the prooxidant elimination of malignant cells without compromising viability and function of healthy bystander cells and tissue.

## Supplementary Materials

The following are available online at https://www.mdpi.com/2072-6694/11/5/590/s1, Figure S1: PMS treatment at concentrations as low as 5 µM impairs viability of human A375 malignant melanoma cells.
