# Supplementary material for: Repurposing the Electron Transfer Reactant Phenazine Methosulfate (PMS) for the Apoptotic Elimination of Malignant Melanoma Cells through Induction of Lethal Oxidative and Mitochondriotoxic Stress
Source: Cancers (Basel). 2019 Apr 27;11(5):590. doi: 10.3390/cancers11050590 (PMC6562717; doi:10.3390/cancers11050590)
Supplement: Supplementary file 1 [file cancers-11-00590-s001.pdf]

Supplementary Materials

Repurposing the Electron Transfer Reactant Phenazine Methosulfate (PMS) for the Apoptotic Elimination of Malignant Melanoma Cells through Induction of Lethal Oxidative and Mitochondriotoxic Stress

Anh B. Hua, Rebecca Justiniano, Jessica Perer, Sophia L. Park, Hui Li, Christopher M. Cabello, and Georg T. Wondrak

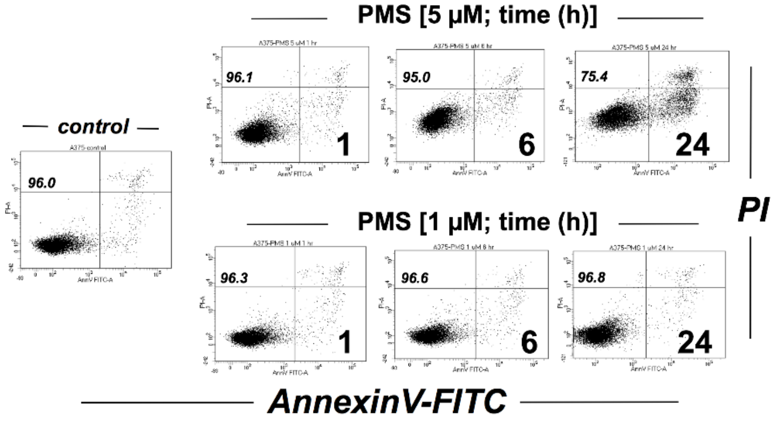

Figure S1. PMS treatment at concentrations as low as 5 μM impairs viability of human A375 malignant melanoma cells. Viability of PMS-exposed cells was monitored using flow cytometric analysis (annexin V-PI staining). Time course of cytotoxicity was examined in PMS-exposed A375 cells (5 μM, 1-24 h, upper row; 1 μM, 1-24 h, lower row). Numbers in quadrants indicate viable (AV-negative, PI-negative) in percent of total gated cells (one specific representative experiment out of three similar repeats is displayed).

© 2019 by the authors. Licensee MDPI, Basel, Switzerland. This article is an open access article distributed under the terms and conditions of the Creative Commons Attribution (CC BY) license (http://creativecommons.org/licenses/by/4.0/).
